# Accelerated clearance of human red blood cells in a rat transfusion model

**DOI:** 10.1186/s40635-015-0064-z

**Published:** 2015-09-18

**Authors:** M. Straat, TRL Klei, D. de Korte, R. van Bruggen, NP Juffermans

**Affiliations:** Department of Intensive Care, Academic Medical Center, Meibergdreef 9, 1105AZ Amsterdam, the Netherlands; Laboratory of Experimental Intensive Care and Anesthesiology, Academic Medical Center, Meibergdreef 9, 1105AZ Amsterdam, the Netherlands; Sanquin Department of Blood Cell Research, Plesmanlaan 125, Amsterdam, the Netherlands

**Keywords:** Transfusion model, Red blood cell, Storage lesion, Red blood cell recovery

## Abstract

**Background:**

Animal models are valuable in transfusion research. Use of human red blood cells (RBCs) in animal models facilitates extrapolation of the impact of storage conditions to the human condition but may be hampered by the use of cross species.

**Methods:**

Investigation of clearance and posttransfusion recovery in a rat model using fresh and stored human RBCs.

**Results:**

Directly following transfusion, human RBCs could be detected in the circulation of all recipients, with higher recovery rates for stored RBCs than for fresh RBCs. After 24 h following transfusion, no donor RBCs could be detected in the circulation, but donor RBCs could be detected in all organs of all recipients.

**Conclusion:**

The use of human donor RBCs in a rat transfusion model resulted in clearance from cells from the circulation. Donor cells were found in different organs of the recipients. Rat transfusion models are thus not appropriate to study the efficacy of human RBC transfusion.

## Background

The growing awareness of transfusion-associated morbidity and mortality has initiated a surge of investigations into the underlying mechanisms, which may be related to storage. Storage lesion of red blood cells (RBCs) includes alteration of the plasma composition, together with changes in morphology of RBCs resulting in reduced deformability, which together may reduce tissue oxygen availability and have pro-inflammatory and immunomodulatory effects [1]. These changes may all reduce posttransfusion viability of red blood cells. For decades, one of the standards of blood conservation as set by the FDA is more than 75 % recovery measured at 24 h posttransfusion [2].

Optimizing storage conditions may negate some of the changes occurring during storage of RBCs. To test hypotheses of storage lesion as well as interventions aimed at improving storage conditions, an appropriate animal model of RBC transfusion would be of great value. However, the biology of stored murine RBCs may not accurately reflect that of human RBCs. Indeed, RBC storage lesion of rodents appears to occur much faster than that of human RBCs. Rodent RBCs stored for 15 days in a commonly used storage solution were reported to rapidly deteriorate, with only 26 % recovery at 24 h following transfusion [3]. However, not all reports using rodent RBCs are consistent, with also reports of normal 24-h posttransfusion survival of stored rodent RBCs [4]. A model in which functional effects of storage can be studied using human RBCs would overcome these problems with translation to the human condition.

To that end, cross species models with transfusion of human RBCs into rodents have been used to study the role of RBC storage in transfusion-related acute lung injury, in recruitment of the microcirculation, in mechanisms of storage lesion, and in rejuvenation of storage solutions [5–7].

However, given the differences in size of the RBCs as well as the capillaries, it is conceivable that the use of cross species may lead to enhanced clearance of human donor RBCs from the rodent circulation. In this study, we investigated the role of storage on routing of human RBCs following transfusion into rodents.

## Methods

The Institutional Animal Care and Use Committee of the Academic Medical Center and the Medical Ethical Committee of Sanquin Blood Bank Foundation approved all experiments. All animals were handled in accordance with the guidelines prescribed by the Dutch legislation and the international guidelines on protection, care, and handling of laboratory animals.

### Preparation of human RBC products

Blood of healthy volunteers was collected using a needle connected to a syringe containing 1.25 ml citrate-phosphate-dextrose. Blood was handled and stored according to national standards for human blood (Sanquin Blood Supply Foundation), by overnight storage at room temperature, followed by centrifugation for 10 min at 1892×*g* and 20 °C. Plasma was removed, and the buffy coat was separated from the packed red blood cells. Saline-adenine-glucose-mannitol was added to the red blood cells up to a hematocrit of 55–60 %. The final products were at 4 °C. Samples of the RBC products were analyzed for pH, potassium, sodium, glucose, and lactate with a Rapidlab 865 blood gas analyzer (Siemens Medical Solutions Diagnostics; Breda, The Netherlands). ATP, 2,3DPG, and hemolysis were analyzed as previously described [8].

### RBC transfusion model

Male Sprague-Dawley rats (275 g) raised on a regular diet were weighed and anesthetized with isoflurane 2 %. Animals were randomized to receive fresh (1 day storage) or stored RBCs (35 days storage, *n* = 6 per group). The tail vein was cannulated with a 24 gauge venflon (Vasofix Certo; B.Braun, Meisungen, Germany), and blood was aspirated to verify intravascular placement. A 10 % circulating volume transfusion was administered. One hour after transfusion, a blood sample was taken. Rats were placed back in their cages to recover. Twenty-four hours after transfusion, rats were sedated with ketamin and medetomidin as described and bled via the inferior caval vein in citrated (0.109 M) vacutainer tubes for analysis and blood culture.

### Assays

Blood taken 1 and 24 h after transfusion were used for cell counts on the Advia 2120 hematology system. Analysis of the human RBC from the circulation was done by spinning down 1 × 10^8^ RBCs for 15 min at 1500 RPM and subsequently removing the top layer containing the white blood cells. RBCs (5 × 10^5^) were washed twice in HEPES and stained with anti-human CD235a-PE (Sanquin Pelicluster Ref M1732) or control antibody (control IgG1PE Ref M1628) and analyzed by flow cytometry (Becton Dickinson LSRII). Data analysis was performed with FACSDiva software (Becton Dickinson).

We were not able to collect organs and analyze them from all animals, because time on flow cytometry and personnel were limited. We measured organs from 3 animals per group. After transfusion, kidney, lung, liver and spleen were collected. The organs were cut into 1–5 mm^3^ cubes, resuspended in HEPES buffer (132 mM NaCl, 20 mM HEPES, 6 mM KCl, 1 mM MgSO_4_, 1.2 mM K_2_HPO_4_) supplemented with 2 mM CaCl_2_ and 10 mM glucose. and homogenized using GentleMACS C-Tubes (Miltenyi Biotec) and the GentleMACS Dissociator (Miltenyi Biotec). Samples were filtered through a 40-μm nylon mesh (Becton Dickinson Cell strainer). Afterwards, 5 × 10^5^ cells were stained with anti-CD235a or control antibody as above.

### Statistical analysis

Results are expressed as mean ± SD. Results of donor RBCs are expressed as percentage human RBCs of the total amount of stained RBCs. Comparison between groups at the same time period or in the same organ was done using Student’s *t* test. A *P* value of <0.05 was considered statistically significant.

## Results

Parameters of storage lesion of the human RBC products are shown in the Table 1.

**Table 1:** Parameters of storage conditions of human RBCs

	RBCs stored 1 day	RBCs stored 35 days
pH	6.96	6.36
Lactate (mmol/L)	5	25
Potassium (mmol/L)	4	22
Glucose (mmol/L)	24	13
ATP (μmol/g Hb)	4	3
2,3DPG (μmol/g Hb)	14.9	0
Hemolysis (%)	0.1	0.124

**Fig. 1:**
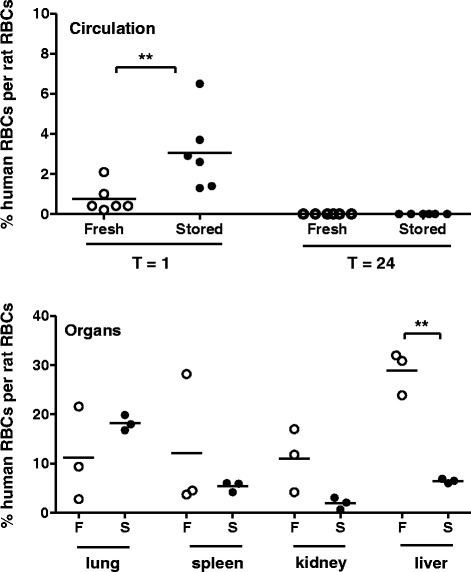
Percentage of human donor RBCs detected in the circulation and in organs of rat recipients, after storage for 1 day (fresh) or 35 days (stored). *F* fresh, *S* stored

Directly following transfusion, human RBCs could be detected in the circulation of all recipients (Fig. 1). Hemoglobin levels were similar to baseline. Of note, the percentage of human RBCs per circulating rat RBCs was higher for stored RBCs than for fresh RBCs.

In contrast, 24 h following transfusion, no donor RBCs could be detected in the circulation, regardless of storage time. Donor RBCs were detected in different organs of all recipients (Fig. 1). The presence of fresh donor RBCs in the liver appeared to be higher compared to stored donor RBCs. Following transfusion, hemoglobin levels were similar to baseline

## Discussion

The use of human donor RBCs in a rat transfusion model resulted in clearance from cells from the circulation, associated with the presence of donor cells in all organs. A possible explanation may be the size of human RBCs, which are somewhat larger than rat RBCs (7–8 versus 6 μm) and may get stuck in the capillaries of organs.

Of note, 1 h following transfusion, we found that the percentage of fresh donor RBCs in the circulation was lower compared to the percentage of stored RBCs, suggesting that fresh RBCs are cleared more rapidly than stored human RBCs. In line with this, the percentage of fresh RBCs stuck in the liver following transfusion was higher compared to stored RBCs. However, this did not hold true for all organs, also not for the lungs, in which the capillaries have the smallest diameter. Besides size, there may be an immunologic mechanism of clearance of human RBCs from the rat circulation. We were unable to define a precise mechanism. Whether fresh RBCs may elicit a stronger immune response than stored RBCs is not known. Alternatively, hemolysis may have played a role. Although we did not measure markers of hemolysis, visual inspection of the samples did not suggest hemolysis.

This study is limited by a small sample size, in particular of the organ measurements, because we were not able to analyze organs from all recipients due to logistic reasons. Although the mechanism is undefined, this study clearly shows that human RBCs get stuck in organs in a rat transfusion model. Given that the standard of appropriate transfusion requires more than 75 % posttransfusion survival, the use of human RBCs in rodent recipients are not appropriate to study the efficacy of RBC transfusion.

## Conclusions

The use of human donor RBCs in a rat transfusion model is not appropriate to study the efficacy of RBC transfusion. Transfusion models should preferably be syngeneic. Thereby, translation of animal storage lesion to the human condition remains a challenge.
